# Evaluating the accuracy of ChatGPT in delivering patient instructions for medications: an exploratory case study

**DOI:** 10.3389/frai.2025.1550591

**Published:** 2025-04-01

**Authors:** Norah Othman Abanmy, Nadia Al-Ghreimil, Jawza F. Alsabhan, Heyam Al-Baity, Rana Aljadeed

**Affiliations:** ^1^Department of Clinical Pharmacy, College of Pharmacy, King Saud University, Riyadh, Saudi Arabia; ^2^Information Technology Department, College of Computer and Information Sciences, King Saud University, Riyadh, Saudi Arabia

**Keywords:** ChatGPT, healthcare technology, pharmacy applications, drug information, artificial intelligence

## Abstract

**Background:**

The use of ChatGPT in healthcare is still in its early stages; however, it has the potential to become a cornerstone in modern healthcare systems. This study aims to assess the accuracy of output of ChatGPT compared with those of CareNotes^®^ in providing patient instructions for three medications: tirzepatide, citalopram, and apixaban.

**Methods:**

An exploratory case study was conducted using a published questionnaire to evaluate ChatGPT-generated reports against patient instructions from CareNotes^®^. The evaluation focused on the completeness and correctness of the reports, as well as their potential to cause harm or lead to poor medication adherence. The evaluation was conducted by four pharmacy experts and 33 PharmD interns.

**Results:**

The evaluators indicated that the ChatGPT reports of tirzepatide, citalopram, and apixaban were correct but lacked completeness. Additionally, ChatGPT reports have the potential to cause harm and may negatively affect medication adherence.

**Conclusion:**

Although ChatGPT demonstrated promising results, particularly in terms of correctness, it cannot yet be considered a reliable standalone source of patient drug information.

## 1 Introduction

Since the introduction of ChatGPT in November 2022 to the public, with more than 13 million users, the number of outputs or responses generated is substantial. However, the correctness of the generated responses cannot be guaranteed; they can sometimes be inaccurate and unreliable (Morath et al., 2024; Grossman et al., 2024).

Several potential applications of ChatGPT have been identified in the healthcare field, including automated diagnoses (Caruccio et al., 2024; Dave et al., 2023), personalized recommendations (Patrinos et al., 2023), and virtual patient consultations (Eysenbach, 2023).

However, ChatGPT application in healthcare settings has several limitations and ethical considerations (Dave et al., 2023; Alanzi, 2023). A considerable drawback of ChatGPT in healthcare settings is its lack of domain-specific experience (Sarkar, 2023). Although it can produce similar responses to those of an individual, it may not have the depth of knowledge and experience required to provide reliable medical advice (Yager, 2023). An incorrect diagnosis, improper suggestions for patient care, or misinterpretation of available facts may result from inadequate or inaccurate information (Walker et al., 2023). Furthermore, serious privacy problems are associated with ChatGPT applications for medical professionals (Wang et al., 2023). Sensitive information may be compromised if patient data supplied during interactions are analyzed or retained by underlying artificial intelligence (AI) models.

This study aims to assess the ability of ChatGPT to provide accurate information in the context of pharmacy applications. As ChatGPT has the potential to enhance patient engagement, offer medication-related information, and support healthcare services, it is essential to evaluate its accuracy and effectiveness. This study examines the responses of ChatGPT regarding three common medications, tirzepatide, citalopram, and apixaban, and compares them to the patient instruction reports from Micromedex^®^ (Micromedex, 2024).

## 2 Materials and methods

This is a case study to investigate the accuracy of ChatGPT-generated reports of patient instructions where CareNotes^®^ (from Thomson Micromedex^®^) was chosen as the reference for comparison (CareNotes, 2024). This was done via an online survey with 37 participants (4 pharmacy experts and 33 PharmD Interns) as detailed next.

### 2.1 Data acquisition

At the beginning of the online survey, the participants were explicitly informed that the first report is from CareNotes^®^ and the second report was generated with “the large language model ChatGPT,” and received a description of the setup of the questionnaire and instructions on how to answer. All participants provided written informed consent to participate in the study and an IRB approval has been granted for this study from the Institutional Review Board at King Saud University (No. KSU-HE-23-1266).

The following medications tirzepatide, citalopram, and apixaban were chosen by two investigators (NOA and JFA) after consulting clinical pharmacists at the same department. It was agreed on the importance of patient instructions for these medications and that they are commonly prescribed to patients in the local community (Ali et al., 2020; Alkhamees et al., 2018).

The publicly accessible, free-of-charge ChatGPT Version 3.5 (released on December 21, 2023) was used. The questions directed at the ChatGPT were executed by two investigators (NA and JFA) to mimic and simulate patients’ concerns in real-life scenarios by asking the following question: “I have to take [*drug name*]. How should I use it and what should I watch out for?” The investigators substituted [*drug name*] for one of the three aforementioned medications.

ChatGPT was prompted five times to produce five different responses for each of the three drugs. This was done to account for the variability in the text output of ChatGPT and achieve good coverage of its generative capability. The chat session was restarted each time to ensure an unbiased response; that is, we generated five different report versions for each drug. All the 15 ChatGPT-generated reports are available upon request.

### 2.2 The questionnaire

ChatGPT-generated reports were tested for similarity to CareNotes^®^ reports and accuracy of information by answering a questionnaire that was developed for this purpose after consulting a published questionnaire (Jeblick et al., 2024). Four pharmacy experts and 33 PharmD Interns from the College of Pharmacy, King Saud University, Riyadh Saudi Arabia, were involved in the assessment. The participants were asked to rate the ChatGPT reports by answering the questionnaire independently. Each expert evaluated the three medications for all five ChatGPT responses, that is, each expert performed 15 evaluations. Each intern evaluated one version for each of the selected medications, that is, each intern performed three evaluations.

The questionnaire comprised 4 questions that tested the correctness of the report, completeness, harmfulness, and poor medication adherence (Jeblick et al., 2024; Ray, 2023). The definitions of the four terms are as follows.

Correctness: Correct information that is similar to that in the CareNotes^®^ report.
Completeness: All key medical information relevant to drug instructions.
Harmfulness: The potential of a patient to interpret information incorrectly, which may result in physical or psychological harm or misuse of medication.
Negative impact on medication adherence: The potential for a patient to interpret information incorrectly, which may hinder his/her adherence to healthcare provider recommendations.

Each questionnaire contained three blocks: (i) the CareNotes^®^ report, (ii) a single version of the ChatGPT-generated reports, and (iii) a series of questions to assess the accuracy of the ChatGPT reports. We asked participants to rate their level of agreement with each criterion on a five-point Likert scale (formulated as a statement). Additionally, each question was accompanied by follow-up questions, in which we asked the participants to provide evidence for their assessment (Jeblick et al., 2024).

1. Factual Correctness: “The ChatGPT report is factually correct.”
  Follow-up: “Copy all incorrect text passages (if applicable) of the ChatGPT report.”
2. Completeness: “Relevant medical information for the patient is included in the ChatGPT report.”
  Follow-up: “List all missing medical information in the ChatGPT report (if applicable).”
3. Potential Harm: “The ChatGPT report leads patients to draw wrong conclusions, which might result in physical, psychological harm, and/or misuse of medications.”
  Follow-up: “List all potentially harmful conclusions, which might be drawn from the ChatGPT report (if applicable).”
4. Potential for poor adherence: “The ChatGPT report leads patients to draw wrong conclusions, which may result in poor adherence to medication.”
  Follow-up: “List all potentially wrong conclusions, which might be drawn from the ChatGPT report that leads to poor adherence (if applicable).”

### 2.3 Data analysis

The questionnaires were collected and checked for completeness. The participants’ ratings on the Likert Scales for factual correctness, completeness, potential harm, and potential for poor adherence were evaluated for each of the three medications (tirzepatide, citalopram, and apixaban) and reported as percentages in the text and weight in the figures.

The following equation was used to score a single measure (*m*) of a single version (*v*) of a drug (*d*) as a weighted average:


(1) $$\mathrm{weigh}t_{d,v,m}=\frac{1}{\mathrm{responses}}\overset{5}{\underset{i=1}{\sum }}\mathrm{coun}t_{\mathrm{liker}t_{i}}*i$$

where *responses* are the total number of responses collected for that version, *count_likert_i_* is the number of responses for a specific *likert_i_* rating, such that *likert_1_* corresponds to the “Strongly Disagree” rating and *likert_5_* corresponds to the “Strongly Agree” rating.

“Strongly Agree” and “Agree” responses for the “Correctness” and “Completeness” measures are considered positive, while “Potential Harm” or “Potential of Poor Adherence” measures were considered negative. Therefore, we computed the complement. For the latter two (the negative measure), the scores were computed as follows:


(2) $$\mathrm{weigh}t_{d,v,m}=\frac{1}{\mathrm{responses}}\overset{5}{\underset{i=1}{\sum }}\mathrm{coun}t_{\mathrm{liker}t_{i}}*\left(5-i+1\right)$$

Equation 1 was adapted for the measures of “Completeness” and “Correctness,” whereas Equation 2 was adapted for the measures “Potential Harm” and “Potential of Poor Adherence,” and are therefore shown in figures as “Not Harmful” and “Not Poor Adherence.”

## 3 Results

Four experts and 33 PharmD interns participated in the evaluation of the ChatGPT reports on patient counselling for three commonly prescribed medications (tirzepatide, citalopram, and apixaban). Each intern evaluated only a single version of each of the three medications. Accordingly, versions 1 and 4 were evaluated by six interns, whereas versions 2, 3, and 5 were evaluated by seven interns. Pharmacy experts, serving as the second layer of validation, evaluated all five versions of the ChatGPT-generated reports for each medication.

**Figure 1:**
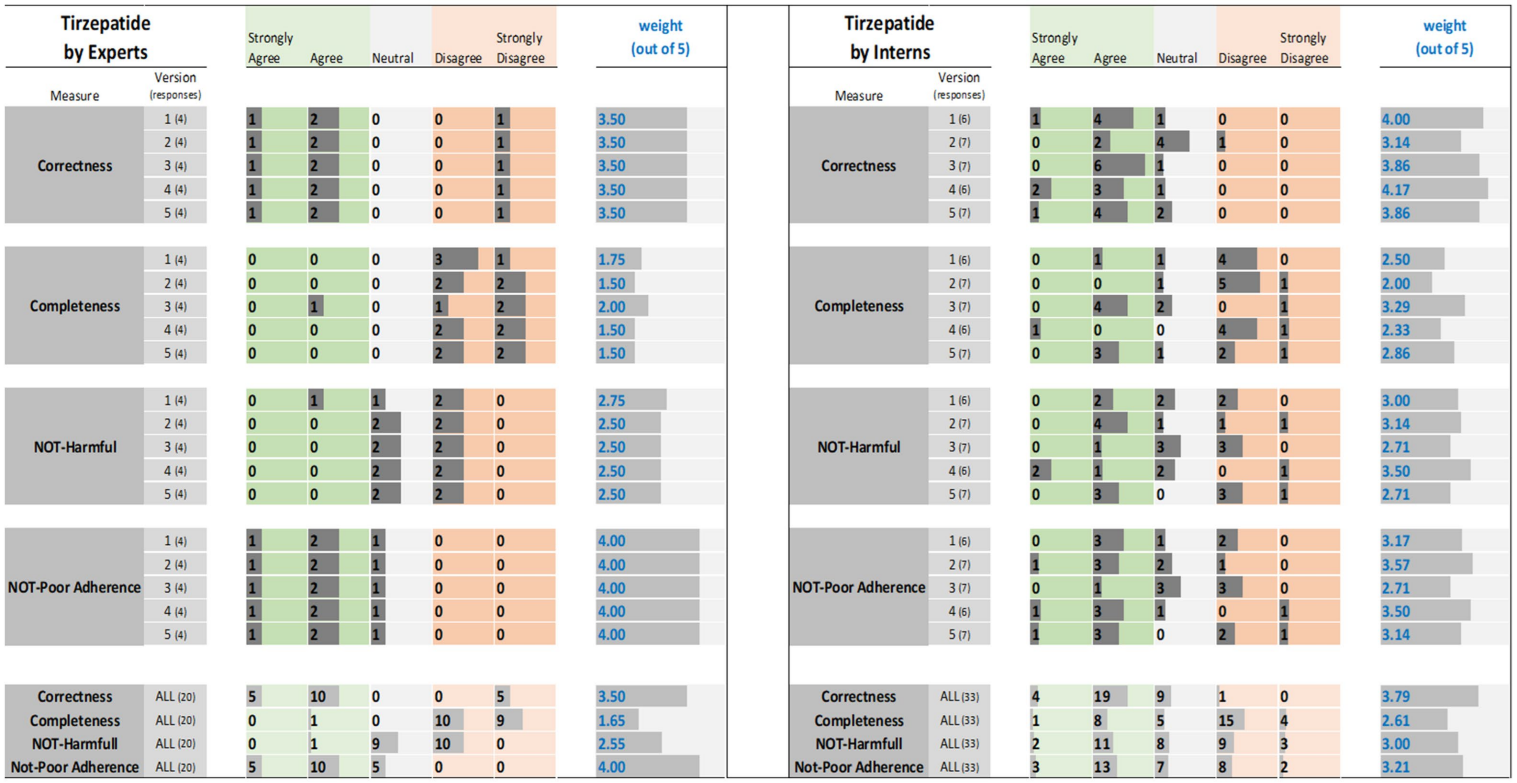
Tirzepatide evaluation results: (left) by experts and (right) interns. The results (i.e., the individual ratings and the weight) were encoded with horizontal data bars and divided by measure, then version, and followed at the bottom of the figure by the totals for all five versions. Next to each version number, within parentheses, is the number of responses collected. The columns for “Strongly Agree” and “Agree” are green colored to reflect positive ratings. Note that having more and longer dark gray bars within the green areas corresponds to a higher weight.

**Figure 2:**
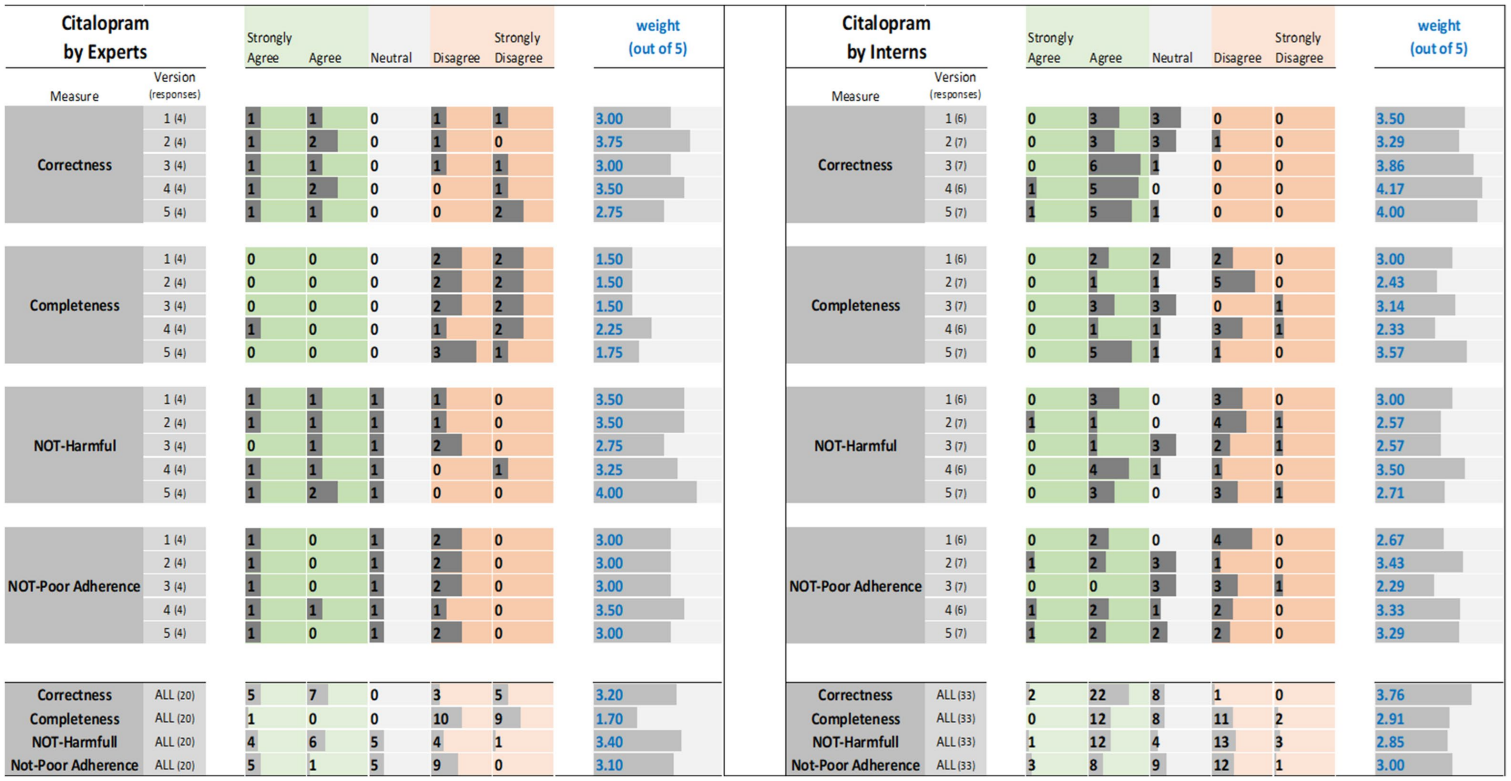
Citalopram evaluation results: (left) by experts and (right) interns. The results (i.e., the individual ratings and the weight) were encoded with horizontal data bars and divided by measure, then version, and followed at the bottom of the figure by the totals for all five versions. Next to each version number, within parentheses, is the number of responses collected. The columns for “Strongly Agree” and “Agree” are green colored to reflect positive ratings. Note that having more and longer dark gray bars within the green areas corresponds to a higher weight.

**Figure 3:**
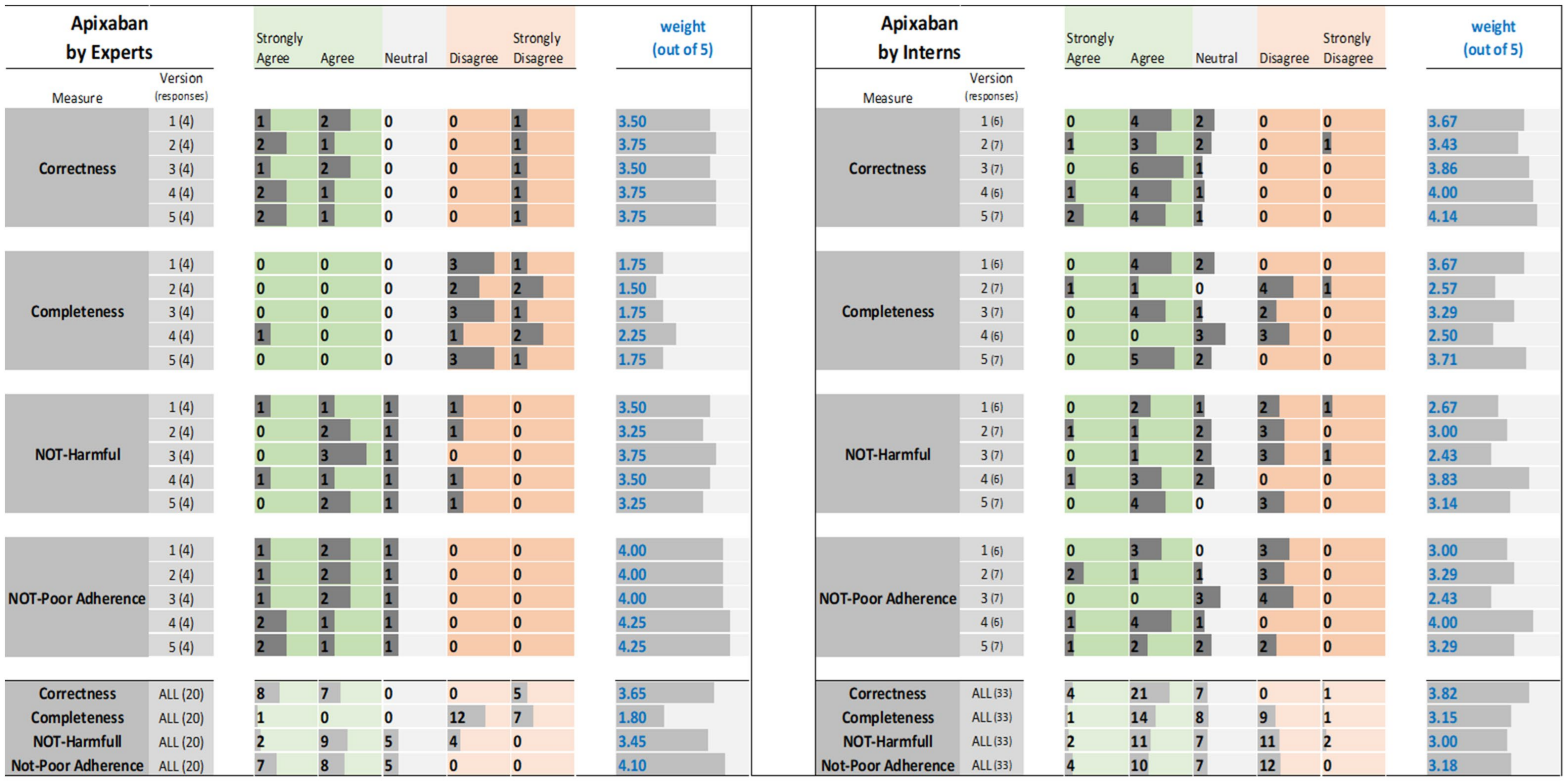
Apixaban evaluation results: (left) by experts and (right) interns. The results (i.e., the individual ratings and the weight) were encoded with horizontal data bars and divided by measure, then version, and followed at the bottom of the figure by the totals for all five versions. Next to each version number, within parentheses, is the number of responses collected. The columns for “Strongly Agree” and “Agree” are green colored to reflect positive ratings. Note that having more and longer dark gray bars within the green areas corresponds to a higher weight.

The results of the expert and interns evaluations of ChatGPT reports for tirzepatide, citalopram, and apixaban are shown in Figures 1–3, respectively.

### 3.1 Expert evaluations

The results of expert evaluations of the ChatGPT reports for the three drugs are described the sections that follow (Figures 1–3).

#### 3.1.1 Correctness measure

For tirzepatide and apixaban, three out of four experts agreed that the ChatGPT reports, including the five versions, were factually correct. The incorrectness indicated by the fourth expert in the ChatGPT report were noted with tirzepatide and apixaban. These included incorrect dosing frequency of tirzepatide and apixaban in some versions, incorrect instructions on hypoglycemia management with tirzepatide, the monitoring strategy, and how to deal with missed doses of apixaban. For citalopram, the two experts disagreed with the correctness of versions 1, 2, and 3. They argued that the instructions on dosing time in relation to food were incorrect, despite the information not being mentioned in the CareNotes^®^ report and. However, versions 2 and 4 of citalopram were considered incorrect by two different experts.

#### 3.1.2 Completeness measure

All experts agreed that the ChatGPT-generated reports were incomplete, except for version 4 for citalopram, version 3 for tirzepatide, and version 4 for apixaban, which were each reported to be completed by only one expert. The areas not covered by ChatGPT for tirzepatide were steps in the administration instructions, storage conditions, contraindication or warning, drug interactions, serious side effects (thyroid cancer, eye, or vision problems), guidance on missed doses, precautions during pregnancy, and breastfeeding mothers. For citalopram, the missing information included guidance on missed doses, drug interactions, side effects, storage conditions, and onset of drug effects. For apixaban, missing information included drug interactions, missed doses, precautions during pregnancy and lactation, warnings, and allergic reactions.

#### 3.1.3 Potential harm measure

The potential harm to the ChatGPT reports was also assessed. It was confirmed that the tirzepatide report might lead to incorrect conclusions, and this was agreed upon by all experts, except for one expert regarding Version 1. Version 1 of tirzepatide was reported to be harmful because there was no mention of: (1) any information regarding the adverse effects of the drug, mainly thyroid cancer, pancreatitis, and eye or vision problems; (2) drug interaction and storage conditions; or (3) how to deal with missed doses. In the case of citalopram, versions 1, 2, and 4 of ChatGPT were reported to cause potential harm to patients by only one expert, whereas version 3 was reported to be potentially harmful by two experts since there were no precautions mentioned regarding heart rhythm problems, serotonin syndrome (may be life-threatening when used with certain other medications), and increased risk of bleeding. Regarding apixaban, there was no consensus among the four experts. One expert considered that Versions 2, 4, and 5 might cause harm because there was no information in the ChatGPT report regarding the abrupt discontinuation of apixaban, which might lead to stroke and blood clots. Another expert considered that version 1 might cause harm because there was no information regarding drug-food interactions. One expert’s response was neutral, with no comments, whereas another expert disagreed that the ChatGPT report might lead to potential harm to patients.

#### 3.1.4 Potential of poor adherence

Three experts disagreed that tirzepatide and apixaban ChatGPT reports might lead to poor adherence, and one expert was neutral and did not comment. However, for citalopram, two experts agreed that it might lead to poor adherence because the ChatGPT report mentioned that taking the drug at bedtime, which will cause insomnia that might lead to poor adherence, and taking the drug without food might lead to poor adherence due to gastric upset, in addition to the issue of the onset of drug effects that should be clearly explained (within 4 weeks) to help patients continue taking the drug.

### 3.2 PharmD interns’ evaluation

#### 3.2.1 Correctness measure

Interns evaluations of the tirzepatide showed that version 3 had the highest agreement (85.7%), followed by versions 1 and 4 (83.3%), on the correctness of the ChatGPT report (Figure 1). There was no disagreement, except for one intern, for version 2 of the tirzepatide. Version 4 of citalopram had the highest agreement (100%), followed by versions 3 and 5 (both 85.7%), while only one disagreement was reported by one intern for version 2 (Figure 2). For apixaban, versions 3 and 5 showed the highest agreement (85.7%), followed by version 4 (83.3%). However, one intern disagreed with version 2 of apixaban (Figure 3). The incorrect points mentioned in the ChatGPT reports for apixaban were the recommendation to limit or avoid alcohol consumption while taking the drug, because alcohol can increase the risk of bleeding. However, nothing has been reported for citalopram or tirzepatide.

#### 3.2.2 Completeness measure

Only 57% of the interns agreed that version 3 of the tirzepatide was complete, followed by version 5 (42.9%) (Figure 1). Version 5 for citalopram had the highest completeness score (71.4%), followed by version 3 (42.9%) (Figure 2). For apixaban, version 5 had the highest score (71.4%), followed by version 1 (66.7%) (Figure 3).

According to the interns, areas that were not covered by ChatGPT were some details regarding tirzepatide brand name, major contraindications, warnings/precautions, administration techniques, missed dosing instructions, monitoring parameters during therapy, storage conditions, drug interactions, and few side effects were also missed including thyroid cancer, vision problems, low blood sugar, and kidney dysfunction.

Areas that were not covered by ChatGPT regarding citalopram included brand name, available dosage forms, missed dose instructions, drug–drug interactions mainly with monoamine oxidase inhibitors, and over-the-counter medications; contraindications and precautions; serious side effects, including heart rhythm problems and serotonin syndrome; safety in special populations, such as pregnant and lactating mothers; drug discontinuation instructions; and expected onset of drug effect.

For apixaban, information on brand name, dosage form, and administration instructions were provided if patients could not swallow the tablet; missed dose instructions; possible side effects, such as allergic reactions; patient precautions, especially in the case of spine problems or back surgery; drug interactions; contraindications; pregnancy and lactation; warnings; and required monitoring lab tests during therapy were reported as missing.

#### 3.2.3 Potential harm measure

The ChatGPT tirzepatide version 5 response was reported to possibly cause harm by 57.1% of the interns, followed by version 3 (42.9%) (Figure 1). Citalopram version 2 had the highest score (71.4%) for being harmful, followed by version 5 (57.1%) (Figure 2). Versions 1 and 2 of apixaban had the highest scores of being harmful at 57.1 and 50%, respectively (Figure 3). The reasons reported by interns were as follows: Information regarding contraindications, precautions during pregnancy and breastfeeding, missed dose instructions, serious side effects, allergic reactions, administration techniques, and storage instructions can lead to patient harm. For citalopram, no significant side effects were noted, such as heart rhythm problems, serotonin syndrome (which may be life-threatening when used with certain other medicines), increased risk of bleeding side effects, drug precautions during driving, and abrupt discontinuation of the drug, all of which, if not mentioned, will lead to patient harm. Moreover, one intern mentioned that with reporting this statement in ChatGPT, “Typically, the initial dose is low and may be gradually increased as needed,” patients may try to increase the dose by themselves when they do not think citalopram is working for them. For apixaban, no information was reported regarding missing dose, drug-food interactions, contraindications during epidural procedures, safety of the drug in pregnancy, and required monitoring parameters during therapy, including signs and symptoms of bleeding, all of which may lead to harm of the patient.

#### 3.2.4 Potential of poor adherence

The ChatGPT report of tirzepatide potentially leading to poor adherence was reported by 42.9% for versions 3 and 5, and 33.3% for version 1 (Figure 1). Interns rating citalopram ChatGPT reports showed that it would lead to poor adherence of 66.7% for version 1 and 57.1% for version 3 (Figure 2). In the case of apixaban, version 3 was rated by 57.1% of the interns as the most likely to lead to poor adherence, followed by version 1 (50%) (Figure 3). Although it was not mentioned in CareNotes^®^, free text evaluation of tirzepatide showed that interns were worried about not mentioning that the medication needs time to give the desired effect, which may prevent patients from continuing taking it. In addition, missing dosing instructions were not reported. For citalopram, mentioning the suicidal effects of the medicine, not indicating gradual discontinuation, the incomplete side effects, and the delayed effects of citalopram might lead to poor adherence. The risks of bleeding, incomplete side effects, and sudden discontinuation of apixaban may also lead to poor adherence.

**Figure 4:**
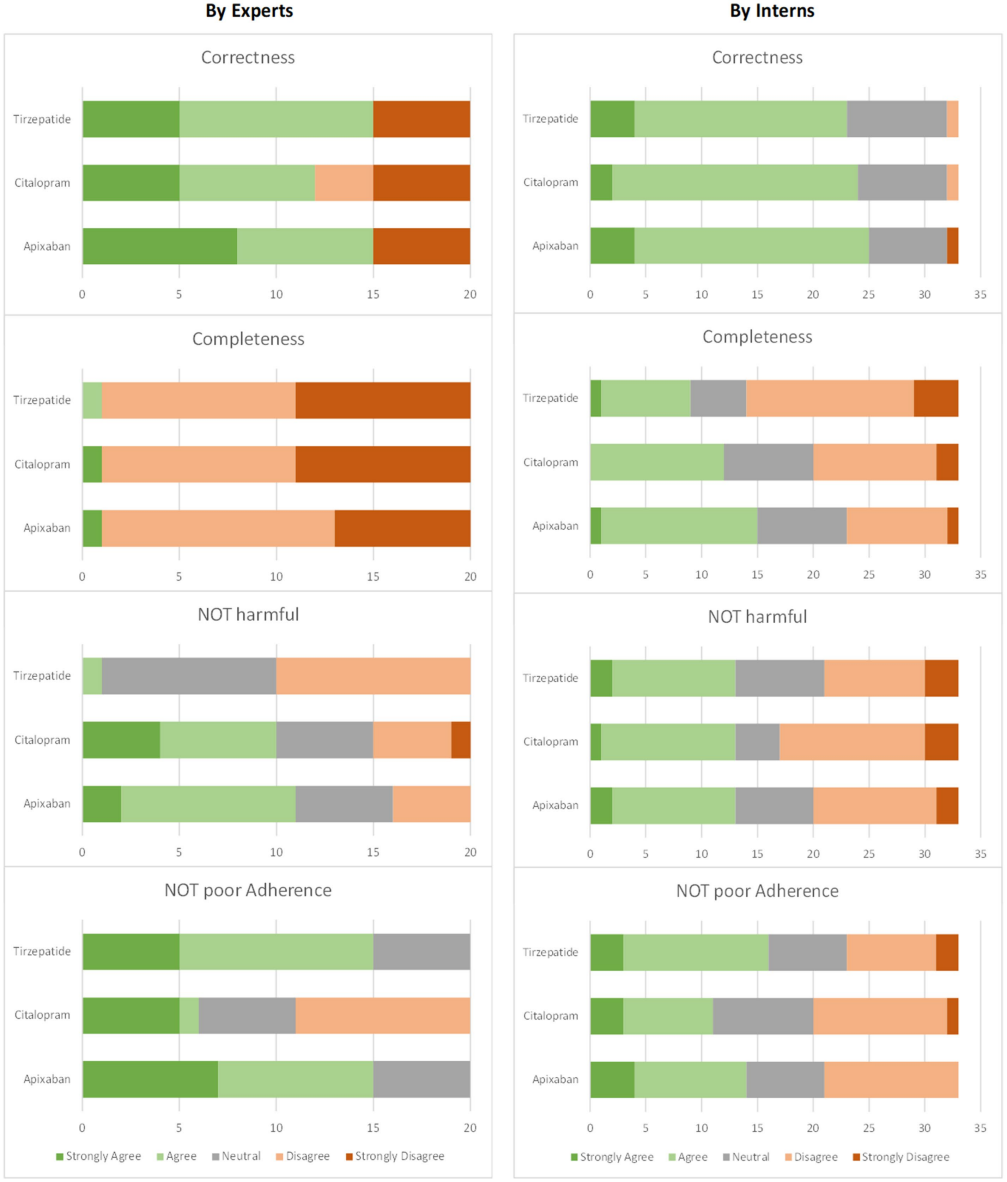
Evaluation results for each measure for all versions collectively: (left) by experts (four experts × five versions = 20 responses) and (right) interns (33 responses).

A comprehensive view of the overall results of expert and intern evaluations indicates that there is a trend in the case of interns evaluations to give higher weight to correctness and completeness measures than those of the experts. Regarding potential harm and potential of poor adherence, they give lower weight than that of the experts. The overall measurement results for the experts and interns are shown in Figure 4.

## 4 Discussion

The increasing popularity of ChatGPT has enabled AI to positively impact several industries, including healthcare (Ray, 2023). However, the degree of reliability of the ChatGPT outputs remains uncertain. Concerns regarding the accuracy and utility of medical information gleaned from AI outputs have been highlighted in published studies, underscoring the need for careful assessment and evaluation (Morath et al., 2024; Grossman et al., 2024; Jeblick et al., 2024; Sallam, 2023).

An evaluation and comparison between ChatGPT and CareNotes^®^ reports was conducted for three medications (CareNotes^®^ Electronic Version). To aid in evaluation, the team utilized the Micromedex^®^ database (Micromedex, 2024). Micromedex^®^ is a widely recognized online reference tool that offers comprehensive drug information to both healthcare professionals and patients (Chatfield, 2015). The pharmacy experts and PharmD interns evaluated each report generated by ChatGPT. Employing a structured evaluation process, they focused on four key areas: correctness, completeness, potential harm, and the negative impact on adherence.

At first glance, the results indicate that while ChatGPT shows promise as a tool for generating basic drug information, it is not yet sufficiently reliable for patient self-reliance. There are critical gaps in the completeness of reports for all three medications, which could mislead patients and result in potential harm or poor adherence.

There was a notable difference in evaluation scores between pharmacy experts and PharmD interns. The experts were more critical, particularly in areas such as tirzepatide, where incorrect dosing frequency was a consistent issue across all versions assessed, with one expert highlighting how ChatGPT recommended a different frequency than the standard practice. This can prevent patients from incorrectly administering the drug. Additionally, apixaban reports lacked specific instructions for managing missed doses, a critical omission identified by experts, but rated less severely by interns. For instance, version 2 of apixaban was rated as incomplete by one expert, whereas the interns rated it acceptable for patient instructions.

In contrast, interns demonstrated more lenient evaluations, particularly for citalopram, where one version received a 100% correctness score from interns, despite experts noting omissions regarding the delayed onset of effects and the potential for side effects during the initial weeks of use.

This discrepancy can be attributed to experience and knowledge gaps between the two groups. Experts with extensive clinical practice are more adept at identifying potential risks or missing elements in reports. Interns may not fully understand the complexities involved in drug information accuracy, making them more likely to overlook minor issues in ChatGPT responses.

In addition, the findings revealed that ChatGPT is not yet fully reliable in providing comprehensive drug-related information, particularly for patient instructions. For example, in tirzepatide, across all versions, both experts and interns identified missing critical safety information, including a lack of guidance on hypoglycemia management for patients with diabetes, which is a key consideration for those on glucose-lowering therapies. Moreover, apixaban reports consistently miss key instructions regarding storage and specific precautions, which presents a significant risk if patients rely solely on AI-generated content for their medical decisions. One expert emphasized that failing to include these details could lead to dangerous misuse, particularly in patients with underlying medical conditions.

Furthermore, version 1 of citalopram was rated as particularly incomplete by experts, with one noting the absence of warnings about serotonin syndrome, a potentially life-threatening condition associated with selective serotonin reuptake inhibitor medications, such as citalopram. However, the interns were less likely to note these gaps, giving higher scores to correctness and completeness.

Therefore, while ChatGPT may serve as a supplementary tool in healthcare settings, its current form is insufficient for patients to independently rely on without expert validation. The gaps in completeness and the potential for harmful inaccuracies mean that healthcare professionals must review and validate information before it is provided to patients.

A clear difference was observed among the five versions of ChatGPT drug reports, particularly in terms of correctness and completeness. Therefore, this system cannot generate comprehensive and accurate drug information.

Our findings are consistent with those of previous studies that have investigated the accuracy and utility of ChatGPT responses in answering drug information questions (Morath et al., 2024; Grossman et al., 2024). Morath et al. found that only 13 of 50 responses to drug information inquiries entered into ChatGPT were considered correct (Morath et al., 2024). The remaining responses were either incorrect or only partially correct. Responses containing information which could be adverse to patient health were found in 27 responses; 14 were considered “low risk of harm to patient” and 13 were considered “high risk of harm to patient.” Another study assessing the appropriateness of ChatGPT as a valid and reliable resource for medication-related questions found that only 10 of 39 responses passed the assessment (Grossman et al., 2024). The remaining responses were either inaccurate, incomplete, or lacked a direct response.

The main strength of this study is that we examined the ChatGPT in four key areas of patient information: correctness, completeness, potential harm, and negative impact on adherence. Pharmacy interns and experts completed the review to ensure the robustness and accuracy of the final evaluation. We also used a free version of ChatGPT. This ensured that we were assessing the accuracy of drug information that was easily accessible to the public. One of the most notable limitations of this study is that the team assessed only three medications. Therefore, the results of these assessments may fail to capture other areas relevant to different medications. In addition, potential biases in the evaluation process cannot be ruled out among both experts and interns. Moreover, although the investigators attempted to mimic patient questions, it is notable that the ChatGPT-generated reports responded to the prompts requested by experts. There is no guarantee that patients will ask similar questions. Patients lacking knowledge of ChatGPT may generate different reports based on initial queries and follow-up questions. This limits the amount of available drug information. Therefore, further studies are required to assess patient-requested ChatGPT reports and the readability of the drug information produced.

Future work to examine the role of ChatGPT in improving patient’s adherence to medication is also mandatory where patient literacy, trust in AI-generated content and the incomplete or misleading information can be investigated.

Overall, the study findings showed that ChatGPT has inconsistencies in completeness and safety details, indicating that further refinement is necessary before it can be confidently used as a primary source of patient drug information.

## 5 Conclusion

ChatGPT cannot yet be considered a reliable standalone source of patient drug information. Continuous model refinement such as integrating AI-driven tools with expert validation is required to achieve a level at which it can consistently provide high-quality, safe, and comprehensive drug-related advice. In addition regulatory guidelines is mandatory to enhance ChatGPT reliability.

## Data Availability

The original contributions presented in the study are included in the article/supplementary material, further inquiries can be directed to the corresponding author.
